# Quercetin Administration Following Hypoxia-Induced Neonatal Brain Damage Attenuates Later-Life Seizure Susceptibility and Anxiety-Related Behavior: Modulating Inflammatory Response

**DOI:** 10.3389/fped.2022.791815

**Published:** 2022-02-11

**Authors:** Yan Wu, Huiping Wei, Pei Li, Hui Zhao, Ruifang Li, Feiyun Yang

**Affiliations:** ^1^Department of Emergency, Hubei Maternal and Child Health Hospital, Tongji Medical College, Huazhong University of Science and Technology, Wuhan, China; ^2^Department of Neurology, Qinghai Provincial People's Hospital, Xining, China; ^3^Department of Neurology, The Third People's Hospital of Hubei Province, Wuhan, China; ^4^Department of Emergency, The First Affiliated Hospital of Xinxiang Medical College, Weihui, China

**Keywords:** hypoxia-induced neonatal seizure, quercetin, inflammation, TLR4, NF-κB

## Abstract

**Background:**

Neonatal seizures commonly caused by hypoxia could lead to brain injury and cognitive deficits. Quercetin could cross the blood brain barrier and exerts neuroprotective effects in many neurological disease settings. In this study, we aim to investigate the role of quercetin in attenuating cognitive impairment following hypoxia-induced neonatal seizure (HINS).

**Method:**

Sprague-Dawley rats at P7 were exposed to a premixed gas in a hypoxic chamber to induce brain injury, and then continuously administered with quercetin for 21 days. Pentylenetetrazol kindling was used to induce seizures in the evolution. After the hypoxic lesion was stablished, anxiety-related behavior of rats after HINS was assessed using open field test. Memory impairment of rats after HINS was evaluated using novel object-recognition test and elevated plus maze test. The serum and hippocampal concentrations of TNF-a, iNOS, IL-6 MCP-1, and IL-1β were measured using ELISA. The mRNA expression levels of TNF-a, iNOS, IL-6 in the hippocampus were determined using qRT-PCR. The protein levels of TLR4, NF-κB p65, and p-NF-κB p65 in the hippocampus were determined using Western blot.

**Results:**

Quercetin administration significantly reduced later-life seizure susceptibility, anxiety-related behavior, and memory impairments in the rats following the HINS when compared to the HINS group without treatment. Both serum and hippocampal proinflammatory cytokines levels were significantly elevated in the rat after HINS. TLR4 protein expressions were increased in the HINS group when compared to control group, and decreased in the group of quercetin. The protein level of p-NF-κB p65 was significantly lower in the quercetin group compared to the HINS group.

**Conclusion:**

We demonstrated that Quercetin significantly reduced susceptibility to later-life seizures. Quercetin could downregulate inflammatory response through TLR4/ NF-κB pathway, thereby attenuating HINS-induced anxiety, hippocampal memory impairment, and cognitive impairment in later life following HINS.

## Introduction

Neonatal seizures are associated with hypoxia damage that occurred within the first 1 or 2 days of life and often could be associated with epilepsy later in life (1). Currently, the occurrence of neonatal seizures is ~0.1–0.5% of newborns and 9–58 per 1,000 very low birth weight infants (2–5). Hypoxic-ischemic encephalopathy, cerebral malformation, and stroke are main contributors to neonatal seizures and commonly are leading to later-life epilepsy and cognitive dysfunction. The fundamental mechanics, however, remain a mystery. Oxidative stress, inflammatory response, and altered ion channel have all been identified to play essential regulatory roles in the development of seizures following brain ischemia (6). Traditional treatment for neonatal seizures such as phenobarbital, phenytoin/fosphenytoin, and levetiracetam have shown almost 50% efficacy, however, side effects including a negative impact on neurodevelopment remain a concern (7–9). Particularly, the anticonvulsant therapy in newborns is unique and may require more evidence. Development and investigation of treatment for neonatal seizures are in urgent need.

Inflammation has been recognized as a major contributor to hypoxia-induced neonatal seizures. The inflammatory response triggered by hypoxia provokes inflammatory cascades, which could result in long-term effects of immune activation and exacerbate brain injury (10). However, the specific mechanism of long-term inflammation impacting epilepsy in later-life of hypoxia-induced neonatal seizures (HINS) remains unclear. A study has shown that the activation of SIRT1 could be responsible for inhibition of TLR4/MyD88/NF-κB signaling pathway thereby reducing neuroinflammation in HINS-induced brain injury (11). These studies highlighted that targeting inflammation could serve as an effective approach of HINS treatment.

It has been reported that quercetin (3, 3′, 4′, 5, 7-pentahydroxyflavone), a flavonoid found in a variety of plants, could cross the blood-brain barrier to reach the brain after systemic administration. Although crossing the blood-brain barrier for flavonoids, particularly Quercetin, may be difficult, many preparations, including nanostructures, have been developed. Quercetin exerts several pharmacological properties, including anti-inflammation and antioxidant, and anti-cancer. Studies have been reported the neuroprotective effects of quercetin in neurological diseases. Sumbul et al. has demonstrated that injection with quercetin showed an anticonvulsant effect in penicillin-induced focal seizure model in adult rats (12). In addition, multiple intraperitoneal treatments of quercetin at a dose of 100 mg/kg significantly increased generalized tonic-clonic seizure onset (GTCS) and decreased GTCS duration when compared to the control (13). A recent study showed that quercetin reduced oxidative stress and TLR4-mediated inflammation in mice with neonatal hypoxic-ischemic brain injury (14). In the present study, we aim to investigate the role of quercetin in reducing later-life seizure susceptibility and anxiety-related behavior in HINS rats.

## Methods

### Rat Model of Neonatal Hypoxia-Induced Seizures

Sprague-Dawley rats were obtained on the 7th day after birth. All rats were housed in cages under the light of 25 ± 2°C and 12/12 h dark/light cycle (humidity was 60–80%) with free access to water and food *ad libitum*. As shown in Supplementary Figure 1, to induce HINS, the rats were exposed to a premixed gas containing 5% O_2_, 95% N_2_ for 15 min in a hypoxic chamber. Animals that exhibited tonic-clonic seizures within 15 min of hypoxia entered the study. Subsequently, animals were exposed to hypoxia or normoxia, and then continuously administered with quercetin for 21 days (Supplementary Figure 2). At P28, behavioral tests were performed. At P42, the pentylenetetrazol (PTZ) was used to assess seizure susceptibility. Briefly, the PTZ was injected intraperitoneally (i.p.) in a sub-convulsant dose of 35.5 mg/kg every other day from P42 with ten injections till P60. Animals were observed for 20 min to assess seizure stage. Tissue and blood were collected for further analysis. The protocols for animal experiments were approved by Hubei Maternal and Child Health Hospital.

### Experimental Design

Rats were randomly divided into four groups: Group 1: Control group treated with sterile physiologic saline (2 mL/kg, intraperitoneally) and 500 IU penicillin (2.5 μl, i.c.), daily for 21 days; Group 2: treated with 25 mg/kg quercetin (2 mL/kg, i.p.) (Sigma-Aldrich; Merck KGaA, Darmstadt, Germany; ≥ 95% (HPLC); dissolved in DMSO, then diluted in 0.9% saline solution) daily for 21 days + 500 IU penicillin (2.5 μl, i.c.); Group 3: treated with 50 mg/kg quercetin (2 mL/kg, i.p.) daily for 21 days + 500 IU penicillin (2.5 μl, i.c.); Group 4: treated with 100 mg/kg quercetin (2 mL/kg, i.p.) daily for 21 days + 500 IU penicillin (2.5 μl, i. c.).

### Open Field Test

The cognitive behavior was evaluated at P60 after hypoxia exposure. To assess motor activity, rats were studied by open field test. The rat was placed in the center of the arena and allowed to explore for 10 min. Total traveled distance and number of central crossing were tracked using video tracking and domestic software (15).

### Novel Object-Recognition Test (NOR)

To evaluate the hippocampal memory function, the NOR test was carried out at P60. The NOR test consists of habituation, familiarity, and novel object recognition testing. These tests were recorded using a camera placed above the open-field box. During the adaptation phase, the rats were allowed to explore the environment without objects for 10 min. In the familiarization phase, an animal was placed in an open field containing two identical objects (A + A) for 10 min. After a retention interval of 10 min, in the test phase, the animal was returned to the field arena with two objects for 10 min, one is the same as the sample and the other is different (A + B). Object record was recorded. The new object index was calculated using the following equation: (B)/(A + B) ^*^ 100. B = spend time with a new object, A + B = spend time with two objects (16).

### Elevated Plus Maze Test (EPM)

The elevated maze consists of two opposite open arms and two opposite closed arms, and a platform. The entire device was fixed at 50 cm above the ground. The rat was placed in the center of the maze, facing one of the closed arms, and was allowed to explore the open or closed arm of the maze for 5 min. The number of entries (“open spare parts”: OAE) and the time spent in open spare parts (“open spare parts time: OAT”) were recorded.

### Seizure Stages Assessment

The animals were observed for 20 min to assess the stage of seizure after each PTZ injection. Seizure stages were determined according to the revised Racine's scale (17). Normal behavior was considered stage 1; hyperactivity was considered stage 2; repeated vertical movements were considered stage 3; forelimb clonus and rearing were considered stage 4; and stage 5, generalized clonic-tonic seizures with fall. Mice scored stage 5 were considered fully kindled in three consecutive PTZ exposures. The median seizure score in each day and the average number of days to reach a fully kindled state were used to assess epileptogenesis. Lastly, myoclonic seizure duration (MS), generalized seizure duration (GS), and latency of stage 5 were measured to evaluate the severity of seizures.

### qRT-PCR

Total RNA was extracted from the hippocampus (8 rats from each group at P28) using Trizol reagent (Invitrogen) according to the manufacturer's protocol. cDNA was primed using cDNA Synthesis Super Mix Kit. The following primers were used: *IL-6* for- ward 5'-GACTGATGTTGTTGACAGCCACTGC−3′, reverse 5′-AGCCACTCCTTCTGTGATCAACT-3′; *TNF-*α, forward 5′- CATGATCCGAGATGTGGAACTGGC-3′, reverse 5′-CTGGCTCAGCCACTCCAC−3′; iNOS, forward 5′- GCATCCCAAGTACGAGTGGT-3′, reverse 5′-GAAGGCGTAGCTGAACAAGG-3′; *GAPDH*, forward 5′-CAAGGTCATCCATGACAACTTTG-3′, reverse 5′- GTCCACCACCCT GTTGCTGTAG-3′.

### Enzyme-Linked Immunosorbent Assay

Serum samples (8 rats from each group at P28) were isolated from the blood after centrifugation at 14,000 rpm for 20 min at 4°C. The concentrations of TNF-a, iNOS, IL-6 MCP-1, and IL-1β in the serum samples or the hippocampus of rats were measured using ELISA kits according to the manufacturer's instructions (BioSource International Inc., Camarillo, CA, USA).

### Western Blot

Proteins were extracted from the hippocampus (8 rats from each group at P28) according to the manufacturer's instructions (KeyGEN, Nanjing, China). The protein concentrations were determined with a modified bicinchoninic acid protein assay kit (KeyGEN). Equal amounts of proteins were loaded and separated by sodium dodecyl sulfate–polyacrylamide gel electrophoresis and then transferred to a polyvinylidene fluoride membrane. The membranes were incubated against TLR4 (#66350-1-Ig, Proteintech), NF-κB p65 (#ab18256, CST), p-NF-κB p65 (#3033, CST), β- actin (#66009-1-Ig, Proteintech) antibodies at 4°C overnight. Horseradish peroxidase-conjugated anti-rabbit or anti-mouse secondary antibodies (Yifeixue, Bio-TECH, Nanjing, China) were then used and blots were imaged with the enhanced chemiluminescence Western blotting Detection System (Millipore). The quantification was conducted using ImageJ software.

### Statistics

Comparisons were conducted by two-way ANOVA followed Tukey's multiple comparisons test and one-way ANOVA followed Dunnett's T3 multiple comparisons test. Data were presented as mean ± SD.

## Results

### Quercetin Administration Following HINS Attenuated Later-Life Seizure Susceptibility in Rats

**Figure 1:**
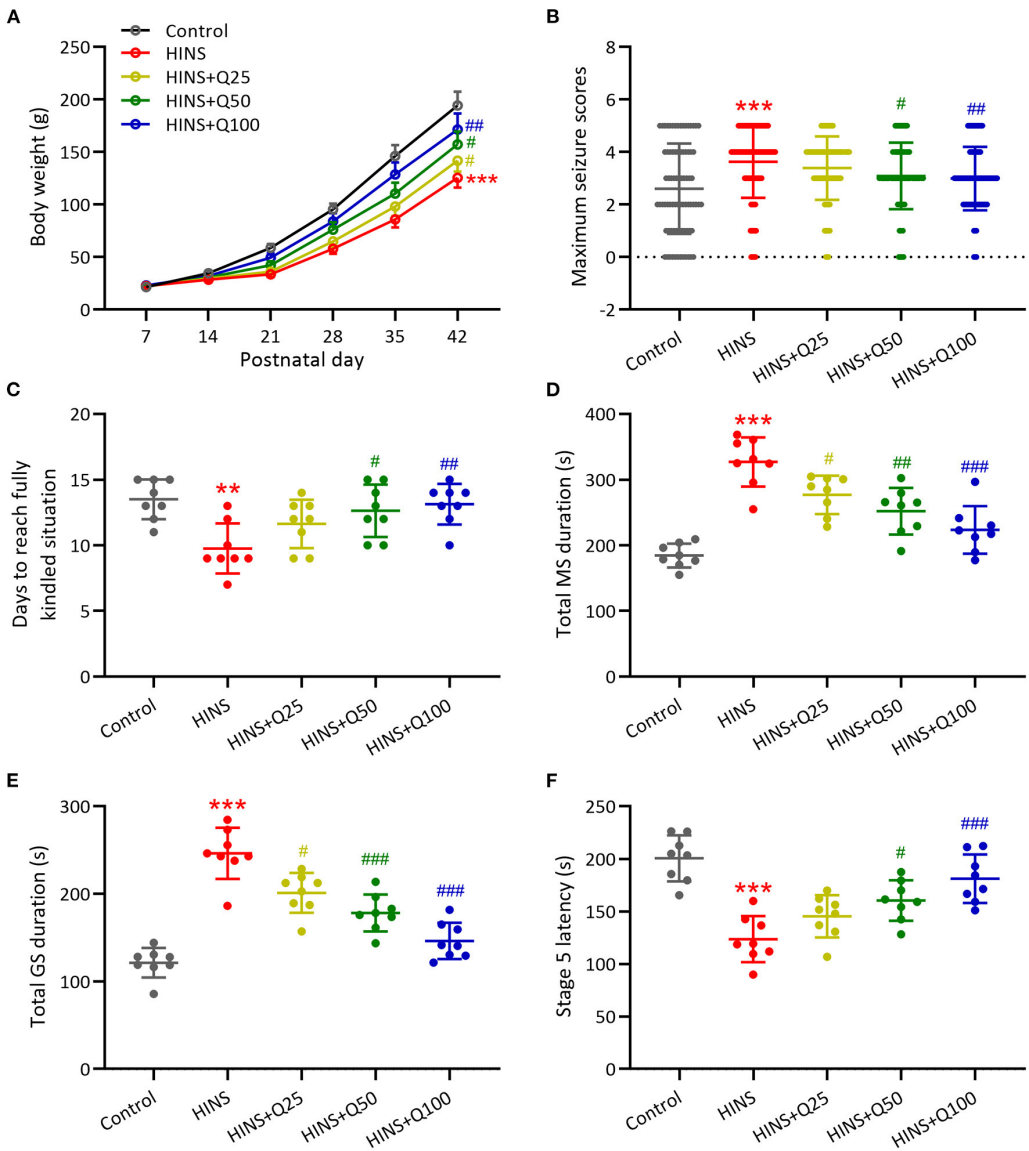
Quercetin administration following HINS attenuated later-life seizure susceptibility in rats. **(A)** Effect of quercetin on body weight following HINS. **(B)** Maximum seizure stages during 20 min after PTZ injections in different experimental groups. **(C)** The averaged days to reach fully kindled situation in different experimental groups. **(D)** Total MS durations were recorded in different experimental groups. **(E)** Total GS durations were recorded in different experimental groups. **(F)** Stage 5 latency were recorded in different experimental groups. Eight rats were used to recorded in each group. Mean ± SD. **p < 0.01, ***p < 0.001 compared to control. #*p* < 0.05, ^##^*p* < 0.01, and ^###^*p* < 0.001 compared to HINS.

To characterize the effects of quercetin administration on HINS in the later life, body weight was measured weekly from P7–P42. Bodyweight gaining was significantly reduced after HINS compared to control group (*p* < 0.001, Figure 1A). Quercetin administration significantly inhibited the loss of body weight in a dose-dependent fashion in comparison with the HINS group at P42 (*p* < 0.01, Figure 1A). Next, to determine whether quercetin administration could affect susceptibility to epileptogenesis after the HINS in the later life, we assessed the maximum seizure stages for 20 min after PTC injections. We found that the maximum seizure scores were remarkedly increased in the HINS group compared with the control group (*p* < 0.001, Figure 1B), whereas significantly reduced by quercetin administration (*p* < 0.001, Figure 1B). Moreover, the average days of fully rekindle in the HINS group were significantly reduced compared to the control group (*p* < 0.01, Figure 1C). In contrast, quercetin notably increased the average days of fully rekindle to that of control (*p* < 0.001, Figure 1C).

In addition, the role of quercetin administration following HINS in the seizure was determined by total MS duration, total GS duration, and stage 5 latency. Our results showed that total MS and GS duration were remarkedly elevated in the HINS group in comparison with control group (*p* < 0.001, Figures 1D,E). In contrast, quercetin administration significantly reduced total MS and GS duration to that of control (*p* < 0.001, Figures 1D,E). Furthermore, the stage 5 latency was significantly decreased in the HINS group when compared to the control group (*p* < 0.001, Figure 1F), which was bounced back to the level of control by quercetin administration (*p* < 0.001, Figure 1F). Taken together, our findings suggested that treatment with quercetin could effectively attenuate later-life seizure susceptibility in HINS rats.

### Quercetin Administration Following HINS Attenuated Anxiety-Related Behavior of Rats

**Figure 2:**
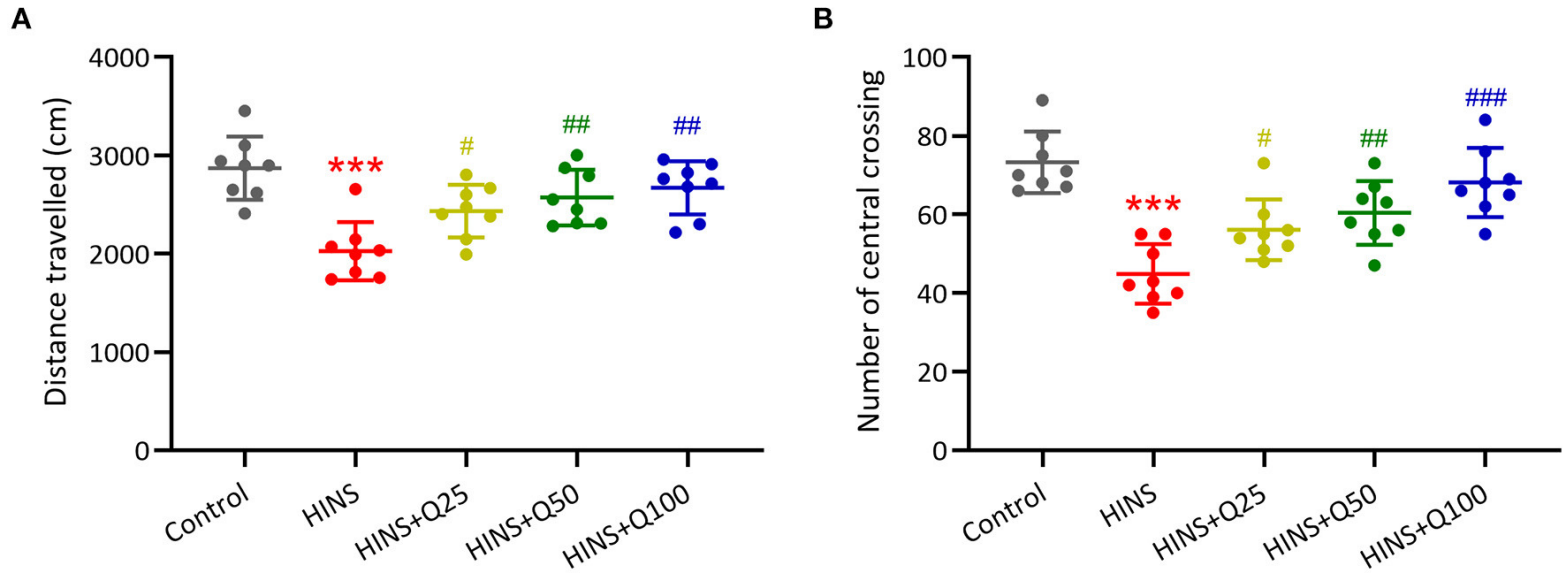
Quercetin administration following HINS attenuated anxiety-related behavior of rats in Open-Field test. Total traveled distance **(A)** and number of central crossings **(B)** over 10 min exploring the arena were recorded. Eight rats were used to recorded in each group. Mean ± SD. ***p < 0.001 compared to control. ^#^*p* < 0.05, ^##^*p* < 0.01, and ^###^*p* < 0.001 compared to HINS.

To determine the effect of quercetin administration on cognitive impairment induced by HINS, we assessed locomotor activity. The traveled distance along with the number of central crossings in the HINS group was significantly reduced when compared to control group (*p* < 0.001, respectively, Figures 2A,B). In contrast, quercetin administration effectively prevented the reduction of traveled distance and the number of central crossings (*p* < 0.01, *p* < 0.001, respectively, Figures 2A,B), indicating the protective role of quercetin in ameliorating HINS-induced cognitive impairment.

### Quercetin Administration Following HINS Attenuated Memory Impairments

**Figure 3:**
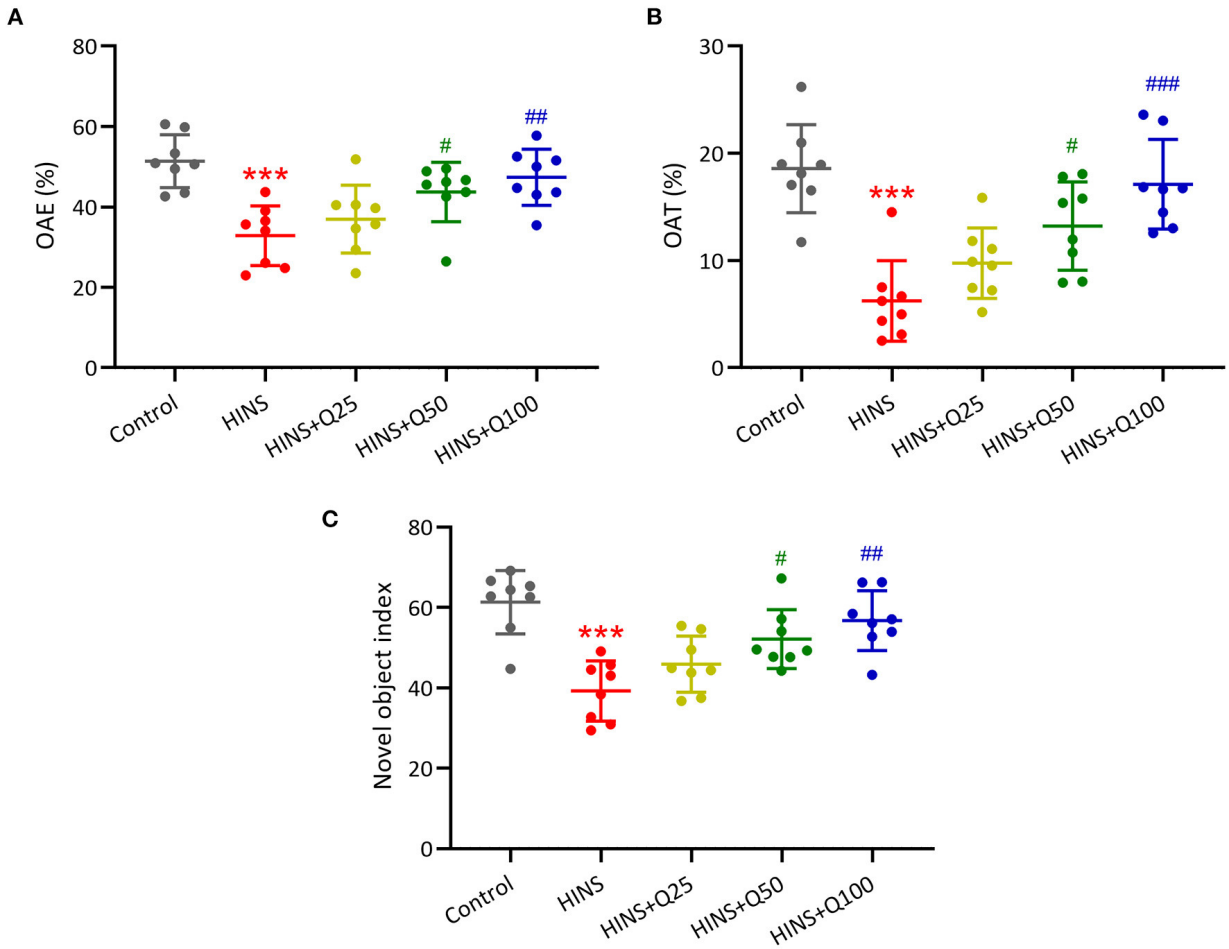
Quercetin administration following HINS attenuated memory impairments of rats in novel object-recognition test (NOR) and elevated plus maze test (EPM). The percentage of OAE **(A)** and OAT **(B)** in EPM were recorded. **(C)** NOR was evaluated by calculating the novel object index. Eight rats were used to record in each group. Mean ± SD. ***p < 0.001 compared to control. ^#^*p* < 0.05, ^##^*p* < 0.01, and ^###^*p* < 0.001 compared to HINS.

Furthermore, to determine the effects of quercetin on HINS-induced memory impairments, we measured memory function using a novel object-recognition test and elevated plus maze test. The percentages of OAE and OAT in the HINS group were remarked decreased when compared to the control group (*p* < 0.001, respectively, Figures 3A,B), which were significantly increased by quercetin administration (*p* < 0.01, *p* < 0.001, respectively, Figures 3A,B). In parallel, the novel object index in the HINS group was significantly decreased relative to control (*p* < 0.001, Figure 3C), which was returned to that of control after quercetin administration in a dose-dependent manner (*p* < 0.01, Figure 3C).

### Quercetin Administration Following HINS Reduced Serum Inflammatory Cytokines

**Figure 4:**
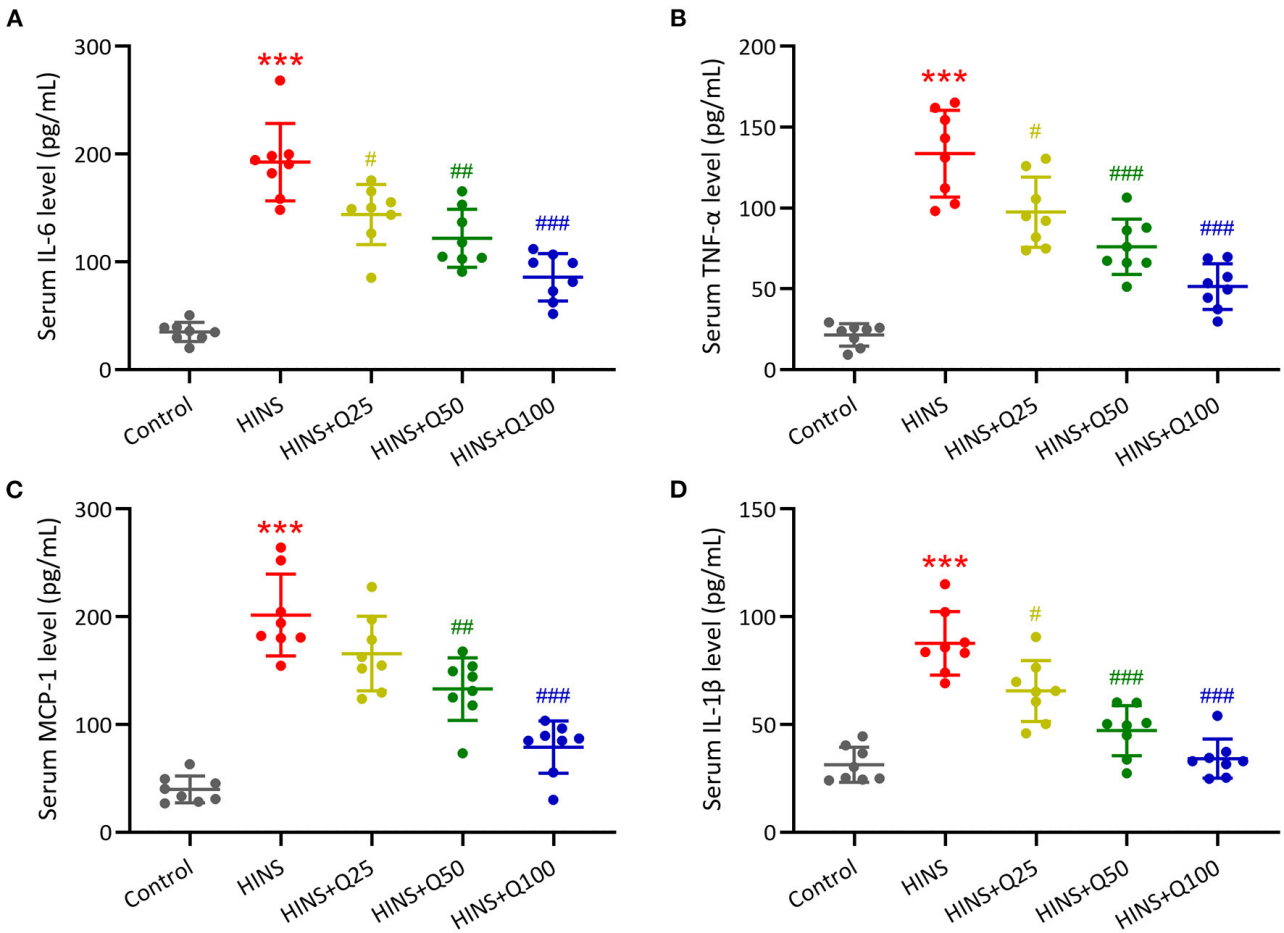
Quercetin administration following HINS attenuated serum inflammatory cytokines. ELISA was used to measure the concentrations of IL-6 **(A)**, TNF-α **(B)**, MCP-1 **(C)**, and IL-1β **(D)** in serum of rats from different groups. Eight rats were used in each group. Mean ± SD. ***p < 0.001 compared to control. ^#^*p* < 0.05, ^##^*p* < 0.01, and ^###^*p* < 0.001 compared to HINS.

To evaluate the role of quercetin in inhibiting inflammatory response, serum inflammatory cytokines IL-6, TNF-α, MCP-1, and IL-1β were measured. Our results showed that the serum levels of IL-6, TNF-α, MCP-1, and IL-1β were dramatically augmented in the HINS group compared to the control (*p* < 0.001, respectively, Figures 4A–D). Quercetin administration significantly reduced the serum levels of IL-6, TNF-α, MCP-1, and IL-1β at P28 in a dose-dependent manner. The serum levels of IL-6, TNF-α, MCP-1, and IL-1β were reduced to the level of control at a dose of 100 mg/kg of quercetin (*p* < 0.001, respectively, Figures 4A–D).

### Quercetin Administration Following HINS Attenuated Hippocampal Inflammatory Response

**Figure 5:**
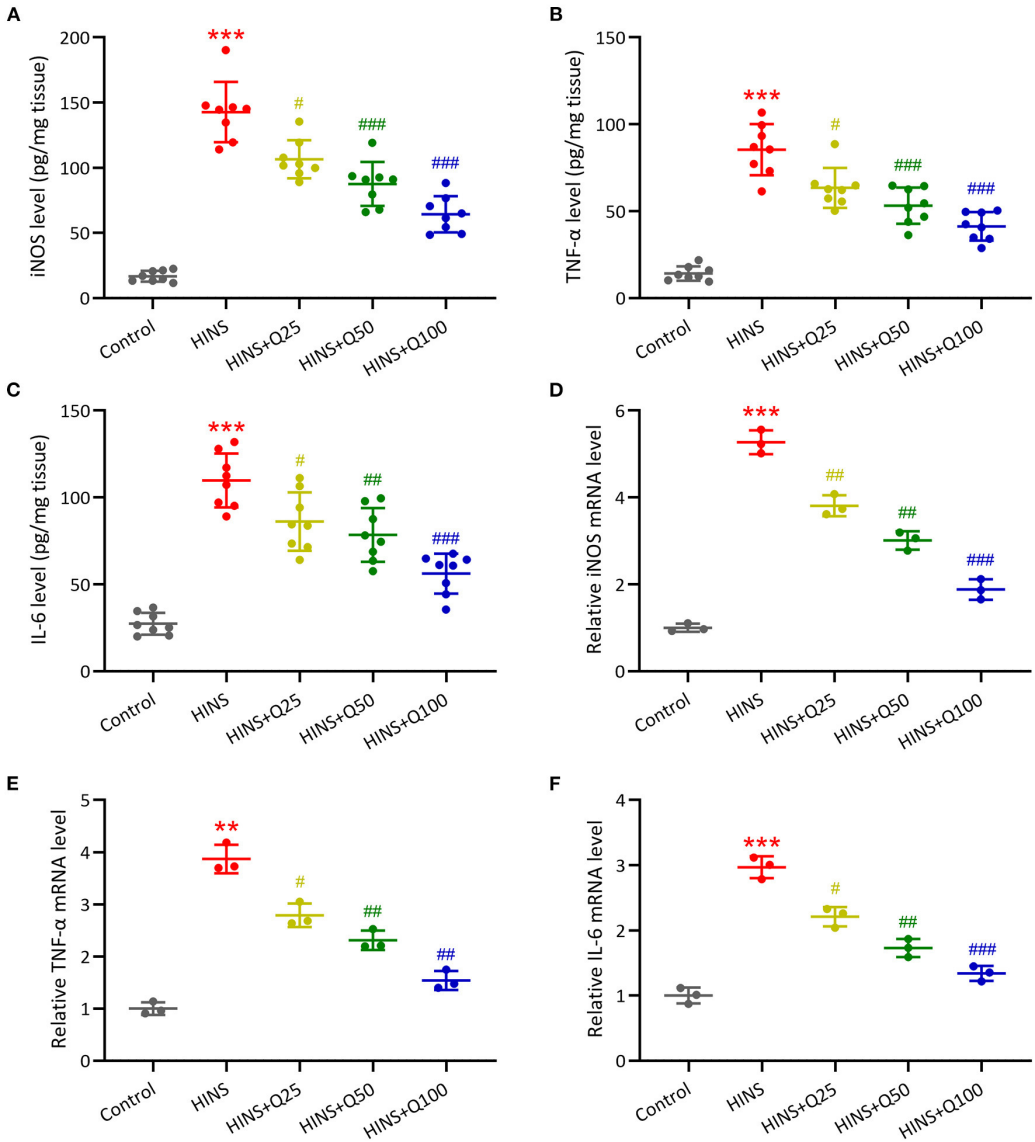
Quercetin administration following HINS attenuated hippocampal inflammatory responses of rats. ELISA was used to measure the concentrations of iNOS **(A)**, TNF-α **(B)**, and IL-6 **(C)** in the hippocampus of rats from different groups. RT-qPCR was used to measure the mRNA expressions of iNOS **(D)**, TNF-α **(E)**, and IL-6 **(F)** in the hippocampus of rats from different groups. Eight or 3 rats were used in each group. Mean ± SD. **p < 0.01, ***p < 0.001 compared to control. ^#^*p* < 0.05, ^##^*p* < 0.01, and ^###^*p* < 0.001 compared to HINS.

To further determine the effect of quercetin on the hippocampal inflammatory response, we measured the concentrations and mRNA expressions of iNOS, IL-6, TNF-α in the hippocampus at P28 using ELISA and RT-qPCR. The concentrations of iNOS, IL-6, TNF-α in the hippocampus were markedly increased in the HINS group compared to the control group (*p* < 0.001, respectively, Figures 5A–C). Notably, treatment with quercetin significantly reduced the levels of iNOS, IL-6, TNF-α in a dose-dependent manner (*p* < 0.001, respectively, Figures 5A–C). Consistent with ELISA results, mRNA levels of iNOS, IL-6, TNF-α were significantly elevated in the HINS group relative to control (*p* < 0.001, respectively, Figures 5D–F) and suppressed by the treatment with quercetin (*p* < 0.001, respectively, Figures 5D–F).

### Quercetin Administration Following HINS Down-Regulated Hippocampal TLR4/NF-κB Signaling

**Figure 6:**
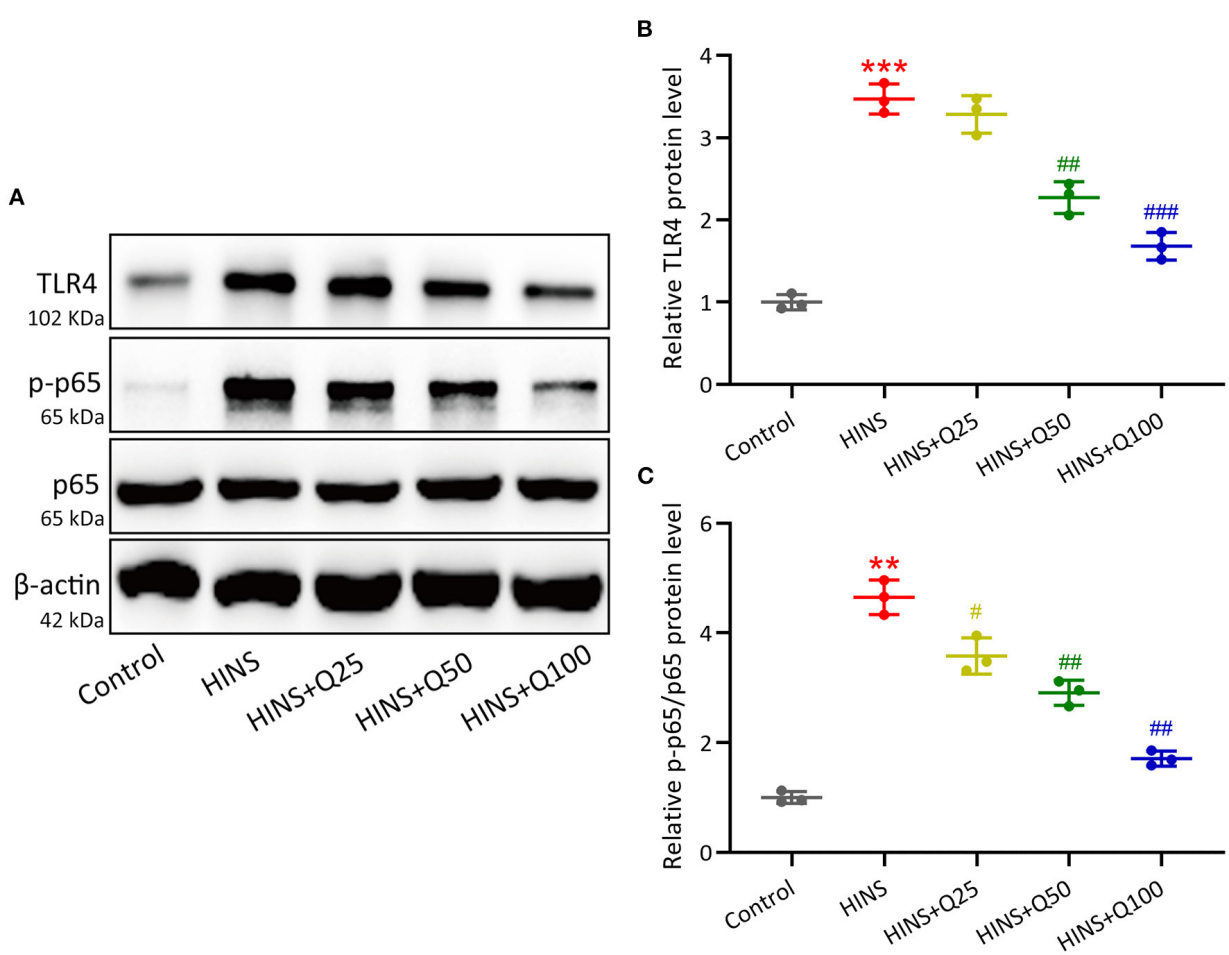
Quercetin administration following HINS down-regulated hippocampal TLR4/NF-κB signaling. The hippocampal protein expressions of TLR4, p-p65, and p65 **(A)** were measured by western blotting. The expressions were normalized to control **(B,C)**. Three rats were used in each group. Mean ± SD. **p < 0.01, ***p < 0.001 compared to control. ^#^*p* < 0.05, ^##^*p* < 0.01, and ^###^*p* < 0.001 compared to HINS.

To evaluate the signaling pathway that is responsible for quercetin in inhibiting inflammatory response during HINS, we assessed hippocampal protein expressions of TLR4/NF-κB using western blot. In the HINS group, the protein level of TLR4 was significantly increased compared to the control group (*p* < 0.001, Figures 6A,B). After quercetin treatment, the level of TLR4 was significantly reduced in a dose-dependent fashion (*p* < 0.001, Figures 6A,B). In parallel, the ratio of NF-κB p-p65 protein to p65 protein was dramatically upregulated in the HINS group when compared to the control group (*p* < 0.01, Figures 6A,C), which was significantly suppressed by quercetin administration (*p* < 0.001, Figures 6A,C).

## Discussion

Cognitive impairment induced by early-life seizures becomes an increasingly appreciated problem. Hypoxic-ischemic brain injury is a major contributor to the development of seizures in neonate; however, the underlying mechanisms are not fully understood. It has been discovered that oxidative stress, inflammatory response, and excitatory-inhibitory neurotransmission imbalance, all play important regulatory roles in the development of seizure after brain ischemic injury. In the present study, we demonstrated that quercetin could change inflammatory response via TLR4/NF-κB signaling, thereby attenuating later-life seizure susceptibility, anxiety-related behavior, and cognitive impairment after HINS in rats.

A population-based study revealed 34% of children developed post-neonatal epilepsy and 41% of children showed neurodevelopmental impairment, implying that high rates of long-term mental impairments and mortality were associated with children with neonatal seizures (18). Notably, a distinct reduction of bodyweight was observed in the rat subjected to HINS, indicating a disability to grow. We found that this effect was significantly abolished by the treatment with quercetin. Najafian et al. demonstrated an increment in seizure susceptibility and hippocampal memory impairment in rats following HINS, which were reduced in both male and female animals by the treatment with fingolimod, a sphingosine 1-phosphate receptor (S1PR) modulator (16). Global hypoxia has been considered as an etiologically related method to provoke neonatal seizures (19). In the present study, P7 rats were used for hypoxia exposure and behavioral seizures in pups with low mortality. In alignment with published works, we showed that HINS in immature rats increased seizure susceptibility to the PTZ kindling model in the later life. Administration with quercetin at different concentrations (25, 50, 100 mg/kg) showed an effectively protective effect in reducing seizure susceptibility to PTZ kindling in the later life of rats with HINS in a dose-dependent fashion. In the HINS group, the increase of MS and GS duration was observed in the HINS group, and treatment with quercetin significantly reduced the duration of MS and GS at all three different concentrations compared to rats with HINS treated with saline. These findings indicate that quercetin has the potential to attenuate seizure susceptibility and anxiety in later life following HINS.

Cognitive deficits such as memory dysfunction and mental disorder are commonly associated with early life seizure. Clinical studies have demonstrated that early-life seizures in children results in significant cognitive dysfunction (20–22). Increasing animal studies provide evidence that early life seizure provokes spatial memory deficits and cellular neuronal injury (23–25). In our study, we examined cognitive impairment in learning and memory in rats following HINS using open file test and elevated plus maze test. Consistent with previous study, behavioral analysis showed that HINS significantly impacted memory and learning function in later life. Notably, quercetin significantly prevented and attenuated cognitive dysfunction in HINS rats. Hippocampus is vulnerable to epilepsy. It has been reported that acute seizures could cause aberrant development in the hippocampus and targeting hippocampal neurogenesis could restore cognitive function after acute epileptic brain insult (26). Hu et al. showed the inhibition of aberrant hippocampal neurogenesis could reduce the susceptibility of epilepsy and cognitive function in rats following HINS (27). In this study, we assessed hippocampal memory function using a novel object recognition test. In agreement with these findings, our data showed the novel object index remarkedly reduced in HINS rats treated with saline, suggesting an impairment in hippocampus-relevant memory function during HINS. Importantly, we found that quercetin administration significantly restored the novel index to that of control, indicating that quercetin effectively improved cognitive function such as hippocampus-relevant memory and learning function.

Inflammation triggered by seizure insult in early life could contribute to hippocampal memory dysfunction and later-life epilepsy. It has been reported that glial activation in early-life results in an acute upregulation of proinflammatory cytokines in the hippocampus could exacerbate long-term neurologic dysfunction (28). In this study, we showed the levels of hippocampal inflammatory cytokines iNOS, IL-6, and TNF-α were dramatically upregulated in rats following HINS and significantly modified by the treatment with quercetin. Quercetin has been shown to inhibit inflammatory cytokines and iNOS by inhibiting the NF-κB pathway in intestinal inflammation (29). Moreover, it has been shown that quercetin downregulated the levels of IL-1β, IL-6, and TNF-α by inhibiting TLR4 and the translocation of NF-κB (30, 31). In our study, we found that the protein expression of TLR4 in the hippocampus was significantly upregulated in HINS rats and decreased in the quercetin treated group. Correspondingly, the hippocampal protein expression of phosphorylated NF-κB p65 markedly increased in HINS rats, whereas significantly reduced by quercetin administration. Taken together, these findings suggest that quercetin inhibit proinflammatory response via TLR4/NF-κB signaling pathway, thereby attenuating cognitive impairment and hippocampal dysfunction in later-life of rats following HINS.

However, there are some limitations in this study. Firstly, due to the small number (*n* = 8) of animals used in the experiment, some of the results of the statistical treatment were not significant in terms of differences. Moreover, in the current study, we couldn't eliminate the influence of penicillin, as it may affect inflammatory response. Therefore, a group without giving penicillin should be included in the future study. Secondly, no other traditional treatment, such as phenobarbital, phenytoin/fosphenytoin, and levetiracetam, were compared. Lastly, future research should include in-depth mechanisms of quercetin in reducing brain damage in HINS.

## Conclusion

Our findings demonstrated that quercetin administration following HINS effectively attenuated later-life seizure susceptibility in rats. In addition, quercetin downregulated the levels of proinflammatory cytokines via suppressing TLR4/NF-κB signaling pathway mechanistically. As a result, quercetin alleviates susceptibility, anxiety-related behavior, and cognitive impairment in later-life of rats following HINS. Our data provide data to support that quercetin could potentially act as an effective approach to prevent and treat HINS.

## Data Availability Statement

The raw data supporting the conclusions of this article will be made available by the authors, without undue reservation.

## Ethics Statement

The animal study was reviewed and approved by Hubei Maternal and Child Health Hospital.

## Author Contributions

FY designed and supervised the study. YW, HW, and PL performed experiments. PL, HZ, and RL analyzed data. YW and HW wrote the manuscript and manuscript revisions. All authors read and approved the final manuscript.

## Funding

This study was supported by science and technology project of Qinghai Province under Grant (no. 2019-ZJ-7101).

## Conflict of Interest

The authors declare that the research was conducted in the absence of any commercial or financial relationships that could be construed as a potential conflict of interest.

## Publisher's Note

All claims expressed in this article are solely those of the authors and do not necessarily represent those of their affiliated organizations, or those of the publisher, the editors and the reviewers. Any product that may be evaluated in this article, or claim that may be made by its manufacturer, is not guaranteed or endorsed by the publisher.

## References

[1] Volpe JJ. Neurology of the newborn. Major Probl Clin Pediatr. (1981) 22:1–648. 7022034

[2] Ronen GM Penney S Andrews W. The epidemiology of clinical neonatal seizures in Newfoundland: a population-based study. J Pediatr. (1999) 134:71–5. 10.1016/S0022-3476(99)70374-4 9880452

[3] Vasudevan C Levene M. Epidemiology and aetiology of neonatal seizures. Semin Fetal Neonatal Med. (2013) 18:185–91. 10.1016/j.siny.2013.05.008 23746578

[4] Andreolli A Turco EC Pedrazzi G Beghi E Pisani F. Incidence of epilepsy after neonatal seizures: a population-based study. Neuroepidemiology. (2019) 52:144–51. 10.1159/000494702 30661067

[5] Padiyar S Nusairat L Kadri A Abu-Shaweesh J Aly H. Neonatal seizures in the U.S. National inpatient population: prevalence and outcomes. Pediatr Neonatol. (2020) 61:300–5. 10.1016/j.pedneo.2019.12.006 31937508

[6] Loscher W Brandt C. Prevention or modification of epileptogenesis after brain insults: experimental approaches and translational research. Pharmacol Rev. (2010) 62:668–700. 10.1124/pr.110.003046 21079040 PMC3014230

[7] Thorp JA O'connor M Jones AM Hoffman EL Belden B. Does perinatal phenobarbital exposure affect developmental outcome at age 2? Am J Perinatol. (1999) 16:51–60. 10.1055/s-2007-993836 10355910

[8] Jensen FE. Neonatal seizures: an update on mechanisms and management. Clin Perinatol. (2009) 36:881–900. 10.1016/j.clp.2009.08.001 19944840 PMC2818833

[9] Favrais G Ursino M Mouchel C Boivin E Jullien V Zohar S. Levetiracetam optimal dose-finding as first-line treatment for neonatal seizures occurring in the context of hypoxic-ischaemic encephalopathy (LEVNEONAT-1): study protocol of a phase II trial. BMJ Open. (2019) 9:e022739. 10.1136/bmjopen-2018-022739 30679288 PMC6347888

[10] Li B Concepcion K Meng X Zhang L. Brain-immune interactions in perinatal hypoxic-ischemic brain injury. Prog Neurobiol. (2017) 159:50–68. 10.1016/j.pneurobio.2017.10.006 29111451 PMC5831511

[11] Le K Chibaatar Daliv E Wu S Qian F Ali AI Yu D. SIRT1-regulated HMGB1 release is partially involved in TLR4 signal transduction: a possible anti-neuroinflammatory mechanism of resveratrol in neonatal hypoxic-ischemic brain injury. Int Immunopharmacol. (2019) 75:105779. 10.1016/j.intimp.2019.105779 31362164

[12] Sumbul O Aygun H. Chronic effects of different quercetin doses in penicillin-induced focal seizure model. Neurosci Lett. (2021) 753:135848. 10.1016/j.neulet.2021.135848 33812925

[13] Nassiri-Asl M Hajiali F Taghiloo M Abbasi E Mohseni F Yousefi F. Comparison between the effects of quercetin on seizure threshold in acute and chronic seizure models. Toxicol Ind Health. (2016) 32:936–44. 10.1177/0748233713518603 24442347

[14] Le K Song Z Deng J Peng X Zhang J Wang L. Quercetin alleviates neonatal hypoxic-ischemic brain injury by inhibiting microglia-derived oxidative stress and TLR4-mediated inflammation. Inflamm Res. (2020) 69:1201–13. 10.1007/s00011-020-01402-5 32944799

[15] Ghafouri S Fathollahi Y Javan M Shojaei A Asgari A Mirnajafi-Zadeh J. Effect of low frequency stimulation on impaired spontaneous alternation behavior of kindled rats in Y-maze test. Epilepsy Res. (2016) 126:37–44. 10.1016/j.eplepsyres.2016.06.010 27423017

[16] Najafian SA Farbood Y Sarkaki A Ghafouri S. FTY720 administration following hypoxia-induced neonatal seizure reverse cognitive impairments and severity of seizures in male and female adult rats: the role of inflammation. Neurosci Lett. (2021) 748:135675. 10.1016/j.neulet.2021.135675 33516800

[17] Van Erum J Van Dam D De Deyn PP. PTZ-induced seizures in mice require a revised Racine scale. Epilepsy Behav. (2019) 95:51–5. 10.1016/j.yebeh.2019.02.029 31026782

[18] Ronen GM Buckley D Penney S Streiner DL. Long-term prognosis in children with neonatal seizures: a population-based study. Neurology. (2007) 69:1816–22. 10.1212/01.wnl.0000279335.85797.2c 17984448

[19] Rakhade SN Klein PM Huynh T Hilario-Gomez C Kosaras B Rotenberg A. Development of later life spontaneous seizures in a rodent model of hypoxia-induced neonatal seizures. Epilepsia. (2011) 52:753–65. 10.1111/j.1528-1167.2011.02992.x 21366558 PMC3071424

[20] Mandelbaum DE Burack GD. The effect of seizure type and medication on cognitive and behavioral functioning in children with idiopathic epilepsy. Dev Med Child Neurol. (1997) 39:731–5. 10.1111/j.1469-8749.1997.tb07374.x 9393886

[21] Mandelbaum DE Bunch M Kugler SL Venkatasubramanian A Wollack JB. Efficacy of levetiracetam at 12 months in children classified by seizure type, cognitive status, and previous anticonvulsant drug use. J Child Neurol. (2005) 20:590–4. 10.1177/08830738050200071001 16159526

[22] Ko A Lee JS. Factors associated with seizure and cognitive outcomes after epilepsy surgery for low-grade epilepsy-associated neuroepithelial tumors in children. Clin Exp Pediatr. (2020) 63:171–7. 10.3345/kjp.2019.01151 32024326 PMC7254172

[23] Koh S Storey TW Santos TC Mian AY Cole AJ. Early-life seizures in rats increase susceptibility to seizure-induced brain injury in adulthood. Neurology. (1999) 53:915–21. 10.1212/WNL.53.5.915 10496246

[24] Karnam HB Zhao Q Shatskikh T Holmes GL. Effect of age on cognitive sequelae following early life seizures in rats. Epilepsy Res. (2009) 85:221–30. 10.1016/j.eplepsyres.2009.03.008 19395239 PMC2795326

[25] Karnam HB Zhou JL Huang LT Zhao Q Shatskikh T Holmes GL. Early life seizures cause long-standing impairment of the hippocampal map. Exp Neurol. (2009) 217:378–87. 10.1016/j.expneurol.2009.03.028 19345685 PMC2791529

[26] Cho KO Lybrand ZR Ito N Brulet R Tafacory F Zhang L. Aberrant hippocampal neurogenesis contributes to epilepsy and associated cognitive decline. Nat Commun. (2015) 6:6606. 10.1038/ncomms7606 25808087 PMC4375780

[27] Hu JJ Yang XL Luo WD Han S Yin J Liu WH. Bumetanide reduce the seizure susceptibility induced by pentylenetetrazol via inhibition of aberrant hippocampal neurogenesis in neonatal rats after hypoxia-ischemia. Brain Res Bull. (2017) 130:188–99. 10.1016/j.brainresbull.2017.01.022 28161194

[28] Somera-Molina KC Robin B Somera CA Anderson C Stine C Koh S. Glial activation links early-life seizures and long-term neurologic dysfunction: evidence using a small molecule inhibitor of proinflammatory cytokine upregulation. Epilepsia. (2007) 48:1785–800. 10.1111/j.1528-1167.2007.01135.x 17521344

[29] Comalada M Camuesco D Sierra S Ballester I Xaus J Galvez J. In vivo quercitrin anti-inflammatory effect involves release of quercetin, which inhibits inflammation through down-regulation of the NF-kappaB pathway. Eur J Immunol. (2005) 35:584–92. 10.1002/eji.200425778 15668926

[30] Byun EB Yang MS Choi HG Sung NY Song DS Sin SJ. Quercetin negatively regulates TLR4 signaling induced by lipopolysaccharide through Tollip expression. Biochem Biophys Res Commun. (2013) 431:698–705. 10.1016/j.bbrc.2013.01.056 23353651

[31] Dong LY Chen F Xu M Yao LP Zhang YJ Zhuang Y. Quercetin attenuates myocardial ischemia-reperfusion injury via downregulation of the HMGB1-TLR4-NF-kappaB signaling pathway. Am J Transl Res. (2018) 10:1273–83. 29887944 PMC5992549

