# Supplementary material for: Quercetin Administration Following Hypoxia-Induced Neonatal Brain Damage Attenuates Later-Life Seizure Susceptibility and Anxiety-Related Behavior: Modulating Inflammatory Response
Source: Front Pediatr. 2022 Feb 11;10:791815. doi: 10.3389/fped.2022.791815 (PMC8873174; doi:10.3389/fped.2022.791815)
Supplement: Supplementary file 1 [file Data_Sheet_1.PDF]

Supplementary materials

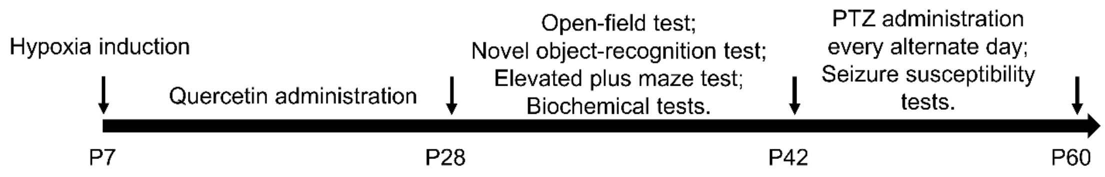

Figure S1. Experimental design flow of the study.

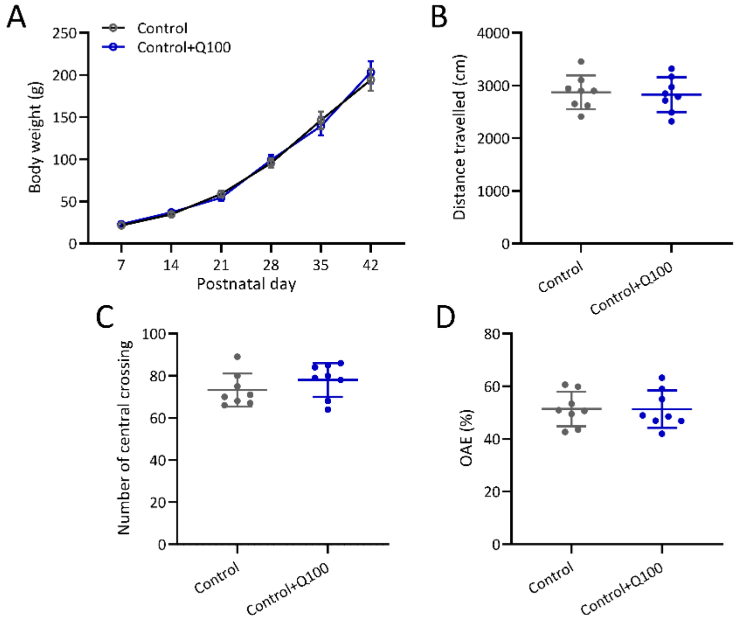

Figure S2. Rats were under the treatment of quercetin at a concentration of 100 mg/kg for three weeks. The body weights were recorded (A). Total traveled distance (B) and number of central crossings (C) over 10 min exploring the arena were recorded in Open-Field test. The percentage of OAE (D) in EPM were recorded. Mean ± SD. No significance between the two groups.
